# Estimating Retinal Sensitivity Using Optical Coherence Tomography With Deep-Learning Algorithms in Macular Telangiectasia Type 2

**DOI:** 10.1001/jamanetworkopen.2018.8029

**Published:** 2019-02-08

**Authors:** Yuka Kihara, Tjebo F. C. Heeren, Cecilia S. Lee, Yue Wu, Sa Xiao, Simone Tzaridis, Frank G. Holz, Peter Charbel Issa, Catherine A. Egan, Aaron Y. Lee

**Affiliations:** 1 Department of Ophthalmology, University of Washington, Seattle; 2 Moorfields Eye Hospital National Health Service Foundation Trust, London, United Kingdom; 3 UCL Institute of Ophthalmology, University College London, London, United Kingdom; 4 Department of Ophthalmology, University of Bonn, Bonn, Germany; 5 Oxford Eye Hospital, Oxford University Hospitals National Health Service Foundation Trust, Oxford, United Kingdom; 6 Nuffield Laboratory of Ophthalmology, Department of Clinical Neurosciences, University of Oxford, Oxford, United Kingdom; 7 eScience Institute, University of Washington, Seattle, Washington

## Abstract

**Importance:**

As currently used, microperimetry is a burdensome clinical testing modality for testing retinal sensitivity requiring long testing times and trained technicians.

**Objective:**

To create a deep-learning network that could directly estimate function from structure de novo to provide an en face high-resolution map of estimated retinal sensitivity.

**Design, Setting, and Participants:**

A cross-sectional imaging study using data collected between January 1, 2016, and November 30, 2017, from the Natural History Observation and Registry of macular telangiectasia type 2 (MacTel) evaluated 38 participants with confirmed MacTel from 2 centers.

**Main Outcomes and Measures:**

Mean absolute error of estimated compared with observed retinal sensitivity. Observed retinal sensitivity was obtained with fundus-controlled perimetry (microperimetry). Estimates of retinal sensitivity were made with deep-learning models that learned on superpositions of high-resolution optical coherence tomography (OCT) scans and microperimetry results. Those predictions were used to create high-density en face sensitivity maps of the macula. Training, validation, and test sets were segregated at the patient level.

**Results:**

A total of 2499 microperimetry sensitivities were mapped onto 1708 OCT B-scans from 63 eyes of 38 patients (mean [SD] age, 74.3 [9.7] years; 15 men [39.5%]). The numbers of examples for our algorithm were 67 899 (103 053 after data augmentation) for training, 1695 for validation, and 1212 for testing. Mean absolute error results were 4.51 dB (95% CI, 4.36-4.65 dB) when using linear regression and 3.66 dB (95% CI, 3.53-3.78 dB) when using the LeNet model. Using a 49.9 million–variable deep-learning model, a mean absolute error of 3.36 dB (95% CI, 3.25-3.48 dB) of retinal sensitivity for validation and test was achieved. Correlation showed a high degree of agreement (Pearson correlation *r* = 0.78). By paired Wilcoxon rank sum test, our model significantly outperformed these 2 baseline models (*P* < .001).

**Conclusions and Relevance:**

High-resolution en face maps of estimated retinal sensitivities were created in eyes with MacTel. The maps were of unequalled resolution compared with microperimetry and were able to correctly delineate functionally healthy and impaired retina. This model may be useful to monitor structural and functional disease progression and has potential as an objective surrogate outcome measure in investigational trials.

## Key Points

**Question:**

Can the probability of retinal sensitivity be estimated from retinal structure seen on commonly used clinical scans (eg, optical coherence tomography) in a retinal disease with a well-defined functional deficit manifesting as a focal blind spot?

**Findings:**

In this cross-sectional study of 2499 microperimetry sensitivities from 63 eyes of 38 patients, deep-learning algorithms estimated retinal sensitivity from optical coherence tomographic scans with a mean absolute error of 3.36 dB.

**Meaning:**

Deep-learning algorithms in this study reliably estimated the outcomes of functional testing with microperimetry, based on optical coherence tomographic scans alone, potentially widening the pool of surrogate markers for vision in clinical practice and therapeutic trials.

## Teaser

This cross-sectional study develops deep-learning algorithms to estimate retinal sensitivity from optical coherence tomographic scans in patients with macular telangiectasia type 2.

## Introduction

A major impediment to the development of novel interventions for rare macular diseases is the lack of sensitive biomarkers that can be used as surrogate clinical trial end points. Particularly when the disease progression is slow, surrogate markers are necessary to overcome the need for large sample sizes and long follow-up duration, which increases the cost and burden of clinical trials and decreases the rate at which new therapeutic interventions can be evaluated. Visual acuity remains a poor functional end point for many macular diseases because the central fovea may be unaffected until late stages of the disease. Paradigmatic for such a disease course is macular telangiectasia type 2 (MacTel), a neurodegenerative disease in which the parafoveal region becomes disproportionately affected compared with the central fovea.^1^

Fundus-controlled perimetry (ie, microperimetry) has the potential to overcome some of these limitations by projecting a localized visual stimulus directly onto exact positions of the patient’s retina. Microperimetry tests visual function outside the central fovea, and thus is better suited for monitoring progression of extrafoveal vision loss, such as in MacTel.^2,3^ Microperimetry can be challenging because of the test duration and the requirement of good fixation and experienced examiners. Despite these challenges, high test-retest reliability has been reported after adequate training of patients and examiners.^4,5,6^ Most acquisition protocols are limited to fewer than 50 spots as a practical compromise between the tolerable test time and the depth and quality of the data. Therefore, subtle losses of sensitivity between the testing areas may be missed. In a clinical trial, the data contained in those areas might make the difference between significant changes of a therapeutic intervention for a slowly progressive disease and a failed treatment trial.

Optical coherence tomography (OCT) is a widely available noninvasive imaging modality capable of providing dense volumetric imaging of the retinal microstructures. Since the advent of OCT, attempts have been made to identify structures on OCT scans that correlate with measured retinal sensitivities in microperimetry. In a conventional structure-function correlation approach, the retinal structure of interest is selected a priori by the researchers, measured, and correlated with the retinal sensitivity at this location.^7^ With this approach, a correlation of ellipsoid zone loss on OCT with microperimetry data has been found.^8,9^ The phase 2 clinical trial of ciliary neurotrophic factor for MacTel was unique for ocular disease in that the US Food and Drug Administration agreed that visual acuity would not be the primary outcome.^10^ Ellipsoid zone loss on OCT was the primary outcome, and functional markers, such as visual acuity, microperimetry, and reading speed, were secondary outcomes. Structures other than the ellipsoid zone may be equally or more suitable as a surrogate measure for retinal function and those structures may vary between diseases. In addition, high-resolution maps of estimated perimetry may provide a functionally more sensitive surrogate end point measure.

Deep learning is a recent advance in artificial intelligence and computer vision in which many layers of convolutional neural networks are stacked to perform end-to-end data-driven learning. Translation of deep-learning tasks into ophthalmic OCT imaging has been mainly limited to classification and segmentation.^11,12,13,14,15,16,17,18,19^ We sought to create a deep-learning network that estimates function from structure de novo using the OCT and microperimetry data to provide an en face high-resolution map of estimated retinal sensitivity.

## Methods

### Image Acquisition

The study population consisted of participants of the Natural History Observation and Registration study of MacTel. The study protocol has been published.^20^ Optical coherence tomographic scans were performed (Spectralis OCT; Heidelberg Engineering) with a volume scan of 97 B-scans.^7^ Microperimetry, which is not part of the standard study protocol, was performed (MAIA; Centervue) using standard grids from the manufacturers as well as a customized testing pattern with a dense central grid with each stimuli 1.5° apart.

The study was reviewed and approved by the regional ethics committees (Ethikkommission der Universität Bonn, Germany, and Health Research Authority Bromley, United Kingdom) and was in adherence to the tenets of the Declaration of Helsinki.^21^ Written informed consent was obtained from each participant. Images were obtained between January 1, 2016, and November 30, 2017, and analyzed between December 1, 2017, and August 15, 2018. Participants did not receive financial compensation. This study followed the Strengthening the Reporting of Observational Studies in Epidemiology (STROBE) reporting guideline.

### Image Processing

The microperimetry results were exported as raw files. Using Adobe Photoshop CS5 12.0 (Adobe Systems Inc), the MAIA infrared scanning laser ophthalmoscope images were superimposed with the Spectralis infrared scanning laser ophthalmoscope images from the volume scan. Retinal vessels were used as landmarks. The image size was changed with constrained proportions and the image was rotated until the vessels were superimposed as closely as possible. No other image distortions were performed.

Spectralis OCT B-scans were selected to intersect the center of each MAIA stimulus. The stimuli intersected by the B-scan were superimposed onto the B-scan aligning their horizontal location on this scan. The resulting B-scan was exported as a .png file with and without superimposing MAIA stimuli. The scans above and below this reference scan were also saved, without a MAIA stimulus.

As described previously,^11^ we created vertical slices of 32 × 496-pixel windows around each perimetry point and used the slice above and below the registered B-scan as additional examples. No other image preprocessing was performed before input into the deep-learning model. No overlapping vertical OCT windows were created to establish the validation and test sets.

### Deep-Learning Approach

Our model is implemented as a deep convolutional neural network with a regression output. To perform regression, a localized vertical slice of the OCT was used as input into the neural network, and the output from the model was set to the microperimetry-measured retinal sensitivity at that location as a continuous variable. By setting the anatomic structure as an input and an objective functional measurement as output, we hoped to create a neural network that could estimate function from structure directly de novo. At inference time, the trained network would provide an en face projection of a high-resolution map of estimated retinal sensitivity.

**Figure 1.**
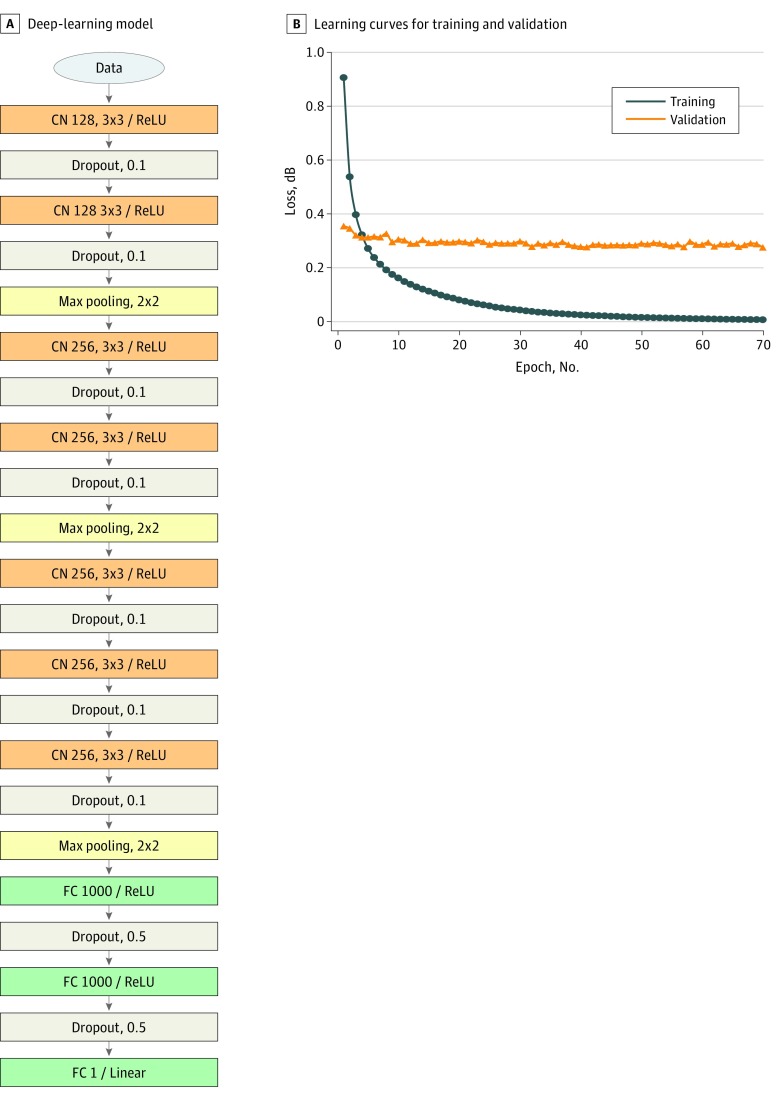
Deep-Learning Model Structure and Learning Curves A, Schematic of deep-learning model using a total of 49.9 million variables in 7 convolutional (CN) and 2 fully connected (FC) layers with rectified linear unit (ReLU) activation. The first number after CN refers to the number of filters and the second set of numbers refers to the filter sizes. The number after FC refers to the number of neurons. Stride was set to 1 on all layers. B, Learning curves for training and validation with mean squared error as the loss function. Note that we applied data augmentation to training data, but not to validation data. Max indicates maximum.

The network architecture is schematically shown in Figure 1A. We used a modified version of the Visual Geometry Group (VGG) 16 convolutional neural network.^22^ The input image is passed through a stack of 3 VGG blocks followed by 2 fully connected layers. The VGG blocks consists of 128, 256, and 256 convolution layers. For better generalization, dropout units were applied to every convolution layer except the last one, with a ratio of 0.1, and each of the fully connected layers with a ratio of 0.5. The total model size was 49.9 million variables.

Weights were initialized using the Xavier algorithm.^23^ The model was trained with mean squared error loss function using Adam optimizer. We chose a batch size of 64. The learning rate was initially set to 1 × 10^−5^, and decay over each update was set to initial learning ratio divided by epochs. All inputs were normalized to a range between 0 and 1, and outputs were normalized to −1 to 1. We assessed the performance of neural network using cross validation with the validation set and evaluated the generalizability with an independent data set. Samples were divided into training sets at 60%, validation sets at 20%, and test sets at 20%. The training, validation, and test sets contained images from mutually exclusive groups of patients (ie, no single patient contributed images to >1 set).

To reduce overfitting on image data, we used data augmentation that consisted of generating image translations, rotations, and horizontal reflections. For translations and rotations, we first identified an approximate position of membrane using Canny edge detector^24^ together with median filter, and vertically shifted and/or rotated the image within an acceptable range. To verify how our deep-learning algorithm predicted the sensitivity, we used Activation Map ^25^ to find spatial support regions for the prediction. Keras^26^ and Python^27^ were used for deep learning. All training occurred using the NVIDIA Pascal Titan X Graphics processing unit with NVIDIA cuda, version 8.0, and cu-dnn, version 5.5.1, libraries.^28^

### Statistical Analysis

All statistical analyses were performed using R, version 3.5.1. The statistical significance level was .05, and 2-tailed paired, nonparametric testing was performed.

## Results

### Network Architecture

The depth of feature maps for each layer was set to 128 in the first block and 256 at the second and third blocks. Lowering the depth of feature maps as 64, 128, and 256 in VGG caused the network to perform worse at evaluation time, and additional depth beyond 256 did not yield further improvements. Making the feature map in the first block deeper did not improve the model performance. Shallower networks, such as LeNet,^29^ caused underfitting of data, and deeper networks, such as VGG16, resulted in overfitting owing to a limited amount of available training images.

### Training, Validation, and Test Results

**Figure 2.**
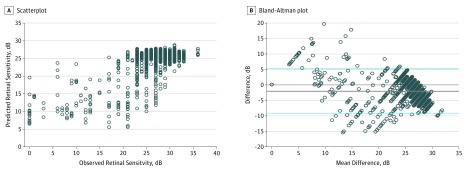
Comparison of Observed vs Estimated Retinal Sensitivity A, Scatterplot showing the observed vs estimated retinal sensitivity. B, Bland-Altman plot showing the difference. The solid gray line represents mean difference obtained across the range of values (mean absolute error), whereas the blue dashed lines are the 95% CIs.

A total of 2499 microperimetry retinal sensitivities were registered to 1708 OCT B-scans from 63 eyes of 38 patients (mean [SD] age, 74.3 years; 15 men [39.5%]). For training, the number of examples available was 67 899 (103 053 after data augmentation); for validation, 1695; and for testing, 1212. After 80 510 iterations per 50 epochs of training, the validation loss stabilized (Figure 1B). The training loss was larger than the validation loss in the first 4 or 5 epochs because the training set included augmented images, which were not included in the validation set. We achieved a mean absolute error of 1.70 dB for training, 3.02 dB for validation, and 3.83 dB for the test set. The model achieved 3.36 dB (95% CI, 3.25-3.48 dB) for the validation and test set. The mean difference determined by Bland-Altman plot was −1.99 dB (95% CI, −9.0 to 5.12 dB) (Figure 2).

### Estimation of Retinal Sensitivity

**Figure 3.**
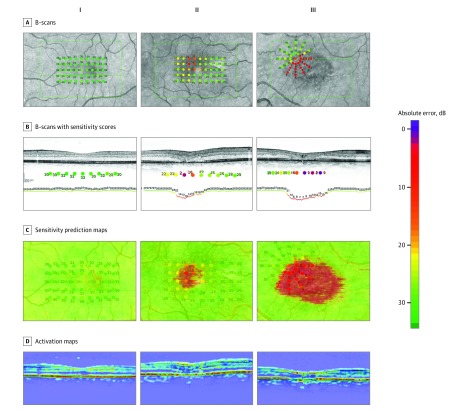
Microperimetry Results and Predicted High-Resolution Sensitivity Maps Three patients with MacTel with varying level of disease severity were chosen from the test set (I, II, III). A, The B-scans that crossed the microperimetry stimuli (green line) were chosen. B, The sensitivity scores were overlaid with those B-scans. The color of those overlaid stimuli was manually corrected, resulting in a discrete color scale for each sensitivity score. After deep learning, the model was able to generate a sensitivity estimation line (below optical coherence tomography B-scan), which showed a good correlation with the observed sensitivities. C, Those prediction lines were stitched together to create high-resolution en face sensitivity estimation maps. D, Activation maps of the B-scan, highlighting the regions that the model used for its estimations. Hot areas (red) corresponded to areas most important for the model to estimate normal perimetry values.

The estimated sensitivity values were used to create high-resolution en face OCT projection maps of estimated retinal sensitivity (Figure 3). After manual registration of the microperimetry to the OCT volume (Figure 3A), the model created an estimate for each vertical line of a B-scan resulting in dense estimation lines for each B scan (Figure 3B). In the next step, those lines were stitched together to en face OCT projection maps (Figure 3C). Our model successfully delineated regions of normal against reduced retinal sensitivities. Figure 3D shows examples of activation map outputs, giving visual explanations from our regression model for which regions on the B scans were important for estimates.

**Figure 4.**
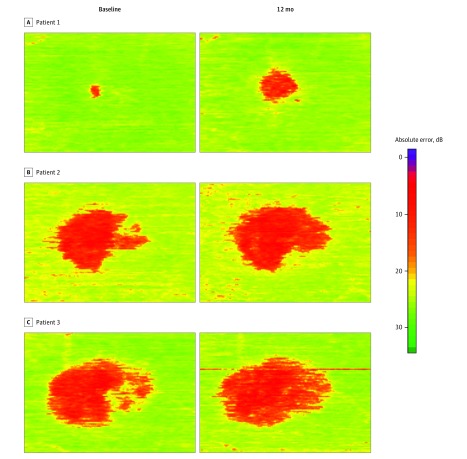
Estimated Progression of Retinal Sensitivity Loss From Longitudinal Optical Coherence Tomographic Scans in 3 Patients With a Follow-up Time of 12 Months Patient 1 (A), patient 2 (B), and patient 3 (C).The area of estimated functional loss (red) increased over time, in keeping with a slowly progressive disorder. These high-resolution estimated perimetry maps indicate that the model might be useful for disease monitoring.

We explored the possibility of applying our method to analyze progression of vision loss in patients with MacTel. For this purpose, we processed OCT volume scans taken approximately 12 months apart (Figure 4). The areas of low retinal sensitivities correlated with each other in location and shape and showed a growth over time that was in keeping with previous studies of natural scotoma progression.^2,8^

**Figure 5.**
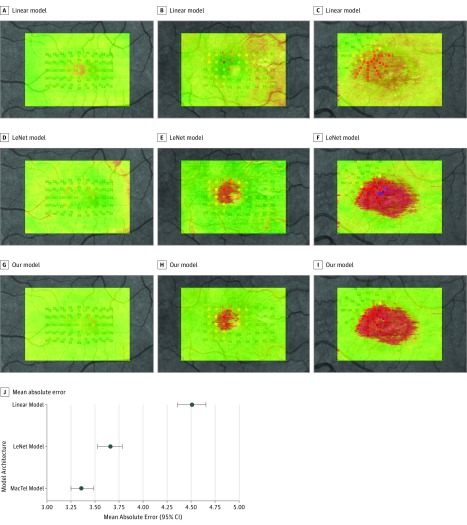
Comparison With Baseline Estimation Models Our model was compared against a linear model as well as the LeNet model. Examples from 3 patients are shown from the test set. A, B, and C represent patients with MacTel with varying degrees of starting disease severity and show the en face estimations for the linear model. D, E, and F, The en face estimations for the LeNet model. G, H, and I, The en face estimations for our model. J, Mean absolute error (95% CIs). Our model achieved significantly better results over these baseline models (*P* < .001, paired Wilcoxon rank sum test).

We compared our model with linear regression and LeNet as baseline models. We trained these models using the same data set and optimization method that we used for our model. Mean absolute error results of these models were 4.51 dB (95% CI, 4.36-4.65 dB) when using linear regression and 3.66 dB (95% CI, 3.53-3.78 dB) when using LeNet. Using a 49.9 million–variable deep-learning model, a mean absolute error of 3.36 dB (95% CI, 3.25-3.48 dB) of retinal sensitivity for validation and test was achieved. Correlation showed a high degree of agreement (Pearson correlation *r* = 0.78). By paired Wilcoxon rank sum test, our model significantly outperformed these 2 baseline models (*P* < .001) (Figure 5).

## Discussion

One of the hallmarks of MacTel is the structural and functional restriction of disease to a specific macular area.^1,30^ Functional loss arises as a sharply delineated scotoma that progresses over time until it reaches a maximum within the MacTel area.^2,31^ This spatial and functional limitation is not only ideal for microperimetry, but also proved to be adaptable to our model, as both diseased and healthy retinal areas are found in the same OCT B-scans, thus offering internal controls. The available sample had a strong left skew with fewer samples of low than high sensitivity scores, affecting the ability of the model to estimate lower sensitivities. Therefore, the variance of deviation between estimated and tested sensitivity was higher for lower tested sensitivities, which might be the reason the estimated areas of functional loss look homogeneous (Figure 3C and Figure 4). However, this deviation was small enough to allow for correct identification of the sharp borders of functional loss and reliably discern healthy from diseased retina (Figure 3B and C).

Microperimetry can be demanding for both patients and examiners. It requires not only time (a correctly performed examination can take 30 minutes for 2 eyes) and intense concentration, but also skilled and motivated examiners and patients, increasing the costs of this method and limiting its use to special cases or questions and selected patient groups. By contrast, an OCT scan requires much less time and minimal patient cooperation. High-quality scans can be obtained in children, adults with disability, and a wide variety of ocular conditions, even with poor visual acuity or poor fixation on a test target. Our approach has the potential of creating dense en face maps of estimated retinal sensitivity, as the OCT scan can cover the retina densely (10-μm interscan distances and widefield scans are commercially available). This high-resolution estimated perimetry map overcomes the natural limitation of every perimetry examination, which can sample only limited areas of the retina; the spatial resolution of the test points, which are limited owing to imprecision of eye tracking and stimulus projections; and the inherent trade-off of an increase in examination duration with lessened patient cooperation for each added test location. These high-resolution maps make the model potentially useful for measuring disease progression (Figure 4); any structural change on OCT scans will be readily translated into functional change by the model.

Several cross-sectional and longitudinal studies have shown a good correlation between retinal function and structure in MacTel, as seen on OCT.^8,9,32,33^ Because those studies only examined the ellipsoid zone (EZ), there might be a potential bias at the expense of other retinal structures that might be functionally more relevant than the EZ, for example, the outer nuclear layer. The model had no human input other than the retinal sensitivity at each microperimetry location, and was therefore unbiased as to which structure to look at on the OCT. The most important structures identified on the activation maps for retinal function were identified within the outer retina in MacTel. The model was able to positively estimate healthy retinal function based on the presence of intact outer retinal layers, in particular, the EZ.

The activation maps provided a close approximation of the en face zones of EZ loss derived from the OCT scans (Figure 3C). Loss of EZ has been recently approved by the US Food and Drug Administration as a primary outcome measure in investigational trials in MacTel (ClinicalTrials.gov identifiers NCT01949324, NCT03316300, and NCT03319849), justified by its correlation to retinal sensitivity measured with microperimetry. Therefore, our model could also be validated and then applied for measurements of surrogate measures in clinical trials of MacTel, as well as another way of monitoring disease progression, because it also takes into account other functionally relevant retinal structures.

Deepening the feature map in the first block did not improve our model performance. High-level structural information captured at a higher layer (second and third blocks of the network) is likely more important for the network to estimate retinal sensitivity than local feature, such as corners and edges captured at the lower layer (first block of the network).

Will this method obviate the need for microperimetry? Such a reliable and validated psychophysical method should not be abolished: retinal function is more meaningful to patients and clinicians than the subtle changes on their retinal scans. Trials are criticized for the use of surrogate end points as markers of therapeutic success; better end points are available. However, many clinics do not have a microperimetry device, whereas OCTs are available almost everywhere. Many patients are unable to perform demanding microperimetry scanning (children, elderly, or disabled patients) and are therefore excluded from clinical trial protocols. Some retinal diseases with wide areas of macular dysfunction are excluded from treatment protocols owing to the lack of a meaningful functional outcome. This model could broaden the inclusion criteria for some of these trials. We chose to focus on MacTel in this proof-of-principle study as a paradigmatic macular disease adaptable to the application of microperimetry owing to the focal and limited nature of its functional loss and anatomic changes.

### Limitations

Although the findings offer the potential to train the model using other more common diseases, such as age-related macular degeneration and diabetic retinopathy, our proof-of-principle study has limitations. This study was based on 1 disease and the findings might not necessarily generalize to all retinal diseases. The precision of the model may improve with additional microperimetry examinations and estimation of the microperimetry from the OCT. Prior work with deep learning and MacTel has created fully automated and reliable approaches for binary segmentation of the EZ band on OCT B-scans.^19^ Our approach differs in that we used a functional measurement as the objective training target without selecting an anatomic feature beforehand.

Future studies could include using dense raster OCT volumes and 3-dimensional convolutions to further increase the model’s accuracy. In addition, with higher-resolution microperimetry testing, U-net style architectures may be used to build deep-learning models for mapping structural OCT to retinal sensitivities.

## Conclusions

We have developed high-resolution en face standard maps of estimated retinal sensitivities with direct relevance to standard microperimetry techniques in eyes with MacTel. The estimates were reliable and fast, and produced similar information compared with microperimetry, correctly delineating functionally healthy and impaired retina. In addition, retinal sensitivity in areas between the test points could be implied from the model. As a proof of principle, we plan to apply this model to other diseases. We believe the model may be useful to monitor structural and functional disease progression and has potential as a surrogate outcome measure in investigational treatment trials.
